# Mental health and wellbeing of further and higher education students returning to face-to-face learning after Covid-19 restrictions

**DOI:** 10.1371/journal.pone.0280689

**Published:** 2023-01-23

**Authors:** Shaun Liverpool, Mohammed Moinuddin, Supritha Aithal, Michael Owen, Katie Bracegirdle, Meggie Caravotta, Rachel Walker, Ciaran Murphy, Vicky Karkou

**Affiliations:** 1 Faculty of Health, Social Care and Medicine, Edge Hill University, Ormskirk, United Kingdom; 2 Centre for Applied Human Rights, University of York, Heslington, United Kingdom; National Cheng Kung University College of Medicine, TAIWAN

## Abstract

**Aim:**

This study aimed to examine the mental health and wellbeing of further and higher education students and the associating factors after returning to face-to-face (in-person) learning after Covid-19 restrictions.

**Methods:**

A cross-sectional study informed by student consultations was conducted using a survey design. Mental health and wellbeing were assessed using self-report items on the Depression, Anxiety and Stress Scale (DASS-21) and the Short Warwick–Edinburgh Mental Wellbeing Scale (SWEMWBS). Descriptive statistics and stepwise multiple linear regression analyses were conducted on data collected between December 2021 and June 2022.

**Results:**

N = 1160 students participated; 69.6% between 16 and 25 years, 67.9% studying in the UK, 66.5% studying away from home, 60.2% identified as she/her, 59.8% studying at the undergraduate degree level, 42.5% belonging to non-White ethnic backgrounds, 29.6% identifying as having additional needs and 22.8% as sexual minority. Moderate anxiety (M = 13.67, SD = 9.92) and depression (M = 17.04, SD = 11.56) scores were mainly reported. Wellbeing scores (M = 20.31, SD = 3.93) were lower than the estimate for the pre-pandemic general population. Gender expression, sexuality, age, ethnicity, having additional needs, and level and location of study was associated with mental health or wellbeing. Individual coping styles, levels of self-efficacy and physical activity were also associated with mental health or wellbeing.

**Conclusions:**

Many students returning to further and higher education after Covid-19 restrictions experienced reduced mental health and wellbeing, and some students were at greater risk. Providing student-centred interventions focusing on self-efficacy, coping styles and physical activity may help improve the mental health and wellbeing of students.

## Introduction

Since the World Health Organization declared the spread of coronavirus (Covid-19) as a pandemic in March 2020 [1], there were several changes in how students accessed further and higher education. Worldwide, colleges, universities and other institutions switched to virtual classrooms and online learning to comply with national and international guidelines [2]. Despite these changes to protect the physical health of students, there was a wealth of evidence suggesting that the mental health and wellbeing of students were negatively impacted. For example, research to date found that symptoms of anxiety and depression were commonly reported among students in pre-degree programmes, as well as undergraduate, graduate, and postgraduate level courses [3–10].

Recent meta-analyses revealed that the average pooled prevalence of anxiety and depression among students ranged from 28% to 40% during the pandemic [3, 5, 10]. Significant factors associated with higher levels of mental distress included studying in low-and-middle-income countries and enrolled in medical programmes. There was mixed evidence for other individual (e.g., age, gender and ethnicity) and programme level (e.g., studying away from home country or region) factors. However, there is wider evidence suggesting that some students were disproportionately impacted. For example, students with disabilities or additional needs were sometimes unable to access their accommodations which lead to varying levels of distress [7, 11, 12]. The disparities in other marginalised communities including sexual and ethnic minority and gender non-conforming groups were also highlighted during the pandemic [13, 14].

In terms of psychosocial correlates, there is growing evidence that self-efficacy of students decreased, and most students reported negative experiences of virtually accessing further and higher education [15–17]. Some experts have attributed these outcomes to increased screen time [18], increased worry beyond Covid-19 [19], less exercise due to closure of gyms [20] and delayed or reduced access to support services [21]. It was also evident that due to the changes in modes of mental health and wellbeing service delivery there have been a risk of dropout and low attendance rates [22]. Despite these challenges, some interventions have been successful in improving mental health and wellbeing [23]. Apart from conventional support, some students also utilised a variety of personal coping strategies [24, 25]. Consequently, longitudinal studies have begun to show improvements in mental health and wellbeing over time to pre-pandemic status [26–28]. However, researchers estimate that around 10% of the population continue to experience distress and these include women and 18-30-year-olds [28–30]. Acknowledging these statistics, over 50% of the university population are typically female and the average age of students fall between the 18 to 30 years age range [31]. Therefore, further explorations are needed to examine mental health and wellbeing recovery trends.

In previous research poor mental health and wellbeing have been associated with poorer academic outcomes and increased dropout from colleges and universities [32]. The impact of poor mental health and wellbeing have also been shown to negatively impact young people’s (16 to 18 years) access to further and higher education [33–35]. Therefore, adequately supporting these students at early stages could help with transitions into further and higher education.

According to government guidelines, a number of academic institutions were allowed to return to in-person teaching and learning during the academic year 2021/2022 [36, 37]. Based on the above evidence it is likely that a large number of students were still experiencing reduced mental health and wellbeing. It is also possible that returning to in-person teaching and learning could have further impacted students’ mental health and wellbeing with the introduction of new factors. Most importantly, there was a need for more research to ensure existing services and interventions are adequate in quantity and quality to support students returning to in-person learning after Covid-19 restrictions.

### Aim and objectives

Primarily this study aimed to examine the mental health and wellbeing of further and higher education students and the associating factors after returning to face-to-face (in-person) learning after Covid-19 restrictions. The main objectives were to 1) provide an overview of the state of students’ mental health and wellbeing when returning to in-person classes, 2) identify groups of students who may be at risk of poorer mental health and wellbeing and 3) identify personal coping strategies that are helpful to students so they can then be incorporated into existing support services or used to inform novel interventions.

## Methods

### Ethical considerations

The Institutional Ethics Review Board at the affiliated university approved the study protocol (ETH2021-0231). Potential respondents were asked to provide digital consent after reviewing the participant information. Participation was fully voluntary, and data were anonymously collected. Confidentiality and privacy were maintained by using unique identification numbers for each case. Participants were assured that the data would only be used for scientific research purposes.

### Participants as co-researchers

The research team consulted students at all stages of the study to ensure this study was carried out with students as lived experience experts and not only for and about students [38]. During the study conceptualisation stage two consultations were held with undergraduate students attending a voluntary value-added learning session. These sessions were optional non-curricula sessions designed to further students’ knowledge and understanding in topic areas related to their primary area of study or interest. Students contributed in several ways. First, students assisted with the construction of the socio-demographic questions and responses. Second, students approved the choice of standardised measures. Third students provided constructive feedback on recruitment materials and dissemination plans. Consultation exercises were facilitated by two of the authors (SA and SL). Outcomes from the sessions are highlighted throughout the manuscript. Further to this, a postgraduate student co-designed the recruitment posters (RW) and two undergraduate students assisted with data collection, data cleaning and data preparation (KB and MC). Consequently, students were also involved in co-authoring this article and therefore contributed to reviewing and editing the manuscript.

### Participant recruitment

This cross-sectional study was conducted in two phases. The survey was piloted with a sample of year 2 undergraduate students (N = 22) at a university in the North-West of England in December 2021 (Phase 1). Thus, data was collected from students almost at the end of their first semester after returning to face-to-face learning at that university, but before the university’s official assessment period. The survey was distributed to a convenient sample of students during a Research Methods seminar hosted by two of the authors (MO and SL). Participation was voluntary and students had the chance to withdraw their data at any point of the study. No significant changes to the survey were required after the pilot phase and the link to the survey was then shared via social media, research platforms, student forums and departmental emails from February 2022 (Phase 2). Participants were also encouraged to share the link with other students. Eligibility criteria was assessed based on age (16 plus years), enrolment at further or higher education institutions (i.e., studying at the Key Stage 5 or 11^th^ Grade and above) and ability to speak and understand English. Therefore, any student meeting the eligibility criteria and encountering the link to the survey had an opportunity to participate in this study.

### Data collection

The average completion time for the survey was between 15 and 20 minutes. The full survey aimed to capture students’ self-report mental health and wellbeing within a six-month period of returning to face-to-face learning after Covid-19 restrictions (https://bit.ly/EHU_Wellbeing). The survey comprised of sociodemographic questions, standardised measures, one modified health behaviour questionnaire and six open ended questions (not included in the current analysis). Further details of the items are provided below.

### Demographic characteristics

Based on guidance from the student consultations, participants were asked to indicate: age as selected from a date calendar; ethnicity by selecting any number of self-identifying options (e.g., White, Black, Asian, Mixed or Other); gender expressions using traditional pronouns (e.g., He/Him); sexuality (e.g., Heterosexual); relationship status (e.g., Single); religion (e.g., Christian); employment status (e.g., Full time) and whether they had any physical, mental or learning difficulties that meant they required additional support (e.g., Yes, No, Unsure, Prefer not to say). Participants were also asked to provide information about their education based on subject (e.g., Arts and Humanities); level (e.g., Undergraduate–Level 4–6); country of study (e.g., UK); enrolment status (e.g., Part time) and whether they were studying away from their home (i.e., responding yes to not living near their family or in the area where they grew up).

### Psychosocial characteristics

#### Mental health

Anxiety and depression were assessed using items on the Depression, Anxiety and Stress Scale (DASS-21, [39]). Each subscale contained seven items. Participants were asked to respond on how closely the item applied to them in the past six months. A Likert scoring system, representing non-conformity (did not apply to me at all, 0) to very consistent (applied to me very much or most of the time, 3) was adopted. The higher the summed score, the higher the level of distress. The DASS subscales for anxiety and depression has shown reliability and validity across different samples [40, 41]. In terms of depression, scores between 0 and 9 were considered normal; 10 to 13 mild; 14 to 20 moderate; 21 to 27 severe and more than 28 extremely severe. For anxiety, scores between 0 and 7 were considered normal; 8 to 9 mild; 10 to 14 moderate; 15 to 19 severe and more than 20 extremely severe.

#### Wellbeing

Wellbeing was assessed using the short version (7 items) of the Warwick–Edinburgh Mental Wellbeing Scale (SWEMWBS). The items were positively worded with five response categories from ‘none of the time’ (1) to ‘all of the time’ (5). The scores on SWEMWBS were summed (range 7–35) with the higher scores relating to better wellbeing. The scores on the SWEMWBS were transformed as per the Warwick conversion table to facilitate statistical analyses. The SWEMWBS has shown reliability and validity in the general population with a mean score ranging 23.2 to 23.7 [42, 43].

#### General self-efficacy

General self-efficacy was assessed using a 10-item self-report scale [44]. This scale has also shown high reliability and validity in previous studies [45, 46]. The participants were asked to confirm how true a list of statements applied to them based on a 4-point Likert scale ranging from ‘not true at all’ (1) to ‘exactly true’ (4). The total score reflected the level of self-perceived general self-efficacy. The total scale score ranges from 10 to 40 with higher scores indicating higher general self-efficacy.

### Potential coping mechanisms

#### Coping styles

The Brief-Cope was used to capture types of coping strategies students adopted during difficult moments. The Brief COPE is a 28-item self-report questionnaire used to assess a number of different coping behaviours and thoughts a person may have in response to a specific situation [47]. The 28-items corresponded to 14 dimensions, each reflecting the use of a coping strategy: active coping, planning, acceptance, denial, self-distraction, use of substance, use of emotional support, use of instrumental support, behavioural disengagement, venting, positive reframing, humour, religion, and self-blame. The items were rated on a Likert scale ranging from ‘I have not been doing this at all’ (1) to ‘I have been doing this a lot’ (4). In this study we adopted the categorisation for this scale that corresponded to problem-focused, emotion-focused, or avoidant coping styles which have been validated in previous studies [48, 49].

#### Physical activity

The extent to which students rated their levels of physical activity was captured using a modified version of the General Practice Physical Activity Questionnaire (GPPAQ) and the corresponding physical activity index. The GPPAQ was validated as a screening tool to be used within the UK National Health Services primary care system for patients over 16 years [50, 51]. In the original screening tool, the type of work done is captured with jobs that required more physical effort (e.g., carpentry, gardening) receiving higher weighting. Based on student consultations, this item was removed as it was anticipated that students may generally be unemployed. Instead, the modified GPPAQ only assessed how much students generally walked, cycled, or engaged with other physical activities (e.g., gym or swimming) during the week. Responses ranged from ‘none’ (1) to ‘3 hours or more’ (4). The total score was then categorised using the physical activity index which represented “moderately inactive (<4)”, “moderately active (5 to 8)” and “active (>9)”. As recommended for measures with less than 10 items the mean interitem correlation was calculated to estimate internal consistency of the modified GPPAQ resulting in an acceptable reliability score of 0.215 [52].

### Data analysis

#### Test of assumptions

Data collected in Phases 1 and 2 were included in the current analysis. There was no significant difference between outcome data collected at the pilot phase and during the main data collection period. Based on inspection of a plot of studentized residuals versus unstandardized predicted values indicated there was homoscedasticity in the data. The histogram and PP-Plot also revealed that the residuals were generally normally distributed. There was also independence of residuals, as assessed by a Durbin-Watson statistic of 1.929. There was no evidence of multicollinearity, as assessed by VIF scores lower than 3.6. Outliers, determined by studentized residuals greater than ±3 standard deviations were not included in the analysis and the mean values for Cook’s distance was below 1 [53].

Patterns of association between the three outcome variables (i.e., anxiety, depression and wellbeing) and the continuous predictor variables were assessed using scatter plots with non-linear loess smoothing. Age was found to be non-linearly associated with all three outcomes. Coping styles also showed a non-linear association with the wellbeing scores. To accommodate these non-linear association pattern in the regression models we used the broken stick regression approach [54]. The break points were determined based on the visual inspection of the loess plot. Analyses were conducted using SPSS 24 [55] and R software version 4.0 [56].

#### Main analysis

Descriptive statistics were calculated using frequencies (%) for categorical variables and central tendencies (Means (M), Standard Deviations (SD)) for continuous variables. During production of the survey the responses to all outcome measures were set as mandatory; therefore, any missing data on the outcome measures were assumed to be missing completely at random. Complete-case analysis (listwise deletion) was adopted to account for missing data. Multiple linear regression (broken stick approach for some variables) analyses were conducted to identify the factors associated with mental health (i.e., anxiety and depression) and wellbeing in students. Factors included demographic (age, gender, ethnicity, first language, sexuality, additional needs, religion, employment status, relationship status), psychosocial (coping styles, level of physical activity, and general self-efficacy) and programme level (studying away from home, level of study, programme of choice, geographic location) characteristics. The significant variables were selected using a stepwise (both forward and backward) approach based on Akaike Information Criterion (AIC). The beta coefficients and their 95% confidence interval are presented using graphs and tables. A two-sided p-value of <0.05 was considered significant. Both the *R*^2^ and adjusted *R*^2^ values are reported.

## Results

### Demographic characteristics of the total sample

A total of N = 1160 students participated in the study. The mean age was 24.85 years (SD = 7.30, Range = 16 to 77 years, 69.6% ≤ 25 years). The majority of the sample (60.2%, n = 689) identified their gender expression as she/her (i.e., traditionally female pronouns) and 3.3% (n = 39) identified as gender non-conforming (i.e., they/them, multiple or other expressions). In terms of ethnicity, the sample was generally representative; 57.5% of the sample identified as White, 21.6% as Black, 8.4% as Asian and 8% as Mixed. The remaining 4.6% indicated Other which included Arab and Latin ethnic backgrounds. Most students identified as heterosexual (77.3%, n = 897), single (47.8%, 554) and not belonging to any religious denominations (47.4%, n = 550). Most students indicated that they did not have any physical, mental or learning difficulties that required additional support (70.4%, n = 817). Most students were also considered to be moderately active (68.8%, n = 798).

Participants were mainly studying in the UK (67.9%, n = 788), Other parts of Europe (7.8%, n = 91), South Africa (15.8%, n = 183) and Mexico (6.2%, n = 72). A smaller number of participants (2.2%, n = 26) were from other countries like Australia, Barbados and Chile. The majority of the students were studying at the undergraduate level of education (59.8%, n = 694), enrolled full time (76.9%, n = 892) and in full time, part time or self-employment (60.3%, n = 700). Programmes relating to Arts and Humanities, Accounts, Business and Management, Engineering and Technology, Life Sciences and Medicine, Natural Sciences and Social Sciences accounted for 86.2% (n = 1000) of the total sample. Most of the students were also native English speakers (74.6%, n = 865) and living in their birth country (82.8%, n = 961) at the time of the study. Taking into account region and country of study, 66.5% (n = 771) of the students were studying away from the area they called *home*. Table 1 provides further details of the total sample.

**Table 1:** Characteristics of the study participants.

Variable	n (%)
Age (years)	
16 to 25	807 (69.6)
26 to 35	253 (21.8)
36 to 77	100 (8.6)
Gender expressions	
He/Him	423 (36.5)
She/Her	698 (60.2)
They/Them	13 (1.1)
Multiple/Other	26 (2.2)
Ethnicity	
White	667 (57.5)
Black	250 (21.6)
Asian	97 (8.4)
Mixed/Multiple	93 (8.0)
Other	53 (4.6)
Country of study	
UK	788 (67.9)
Rest of Europe	91 (7.8)
South Africa	183 (15.8)
Mexico	72 (6.2)
Other	26 (2.2)
Sexuality	
Heterosexual	897 (77.3)
Bisexual	153 (13.2)
Homosexual	55 (4.7)
Other/Unsure/Prefer to say	55 (4.7)
Have additional needs	
No	817 (70.4)
Yes	229 (19.7)
Unsure	89 (7.7)
Prefer not to say	25 (2.2)
Physically active	
Moderately active	798 (68.8)
Active	225 (19.4)
Moderately inactive	137 (11.8)
Level of study	
Level 1–3 (e.g., 6^th^ form college)	174 (15.0)
Level 4–6 (e.g., Undergraduate)	694 (59.8)
Level 7–8 (e.g., Postgraduate)	292 (25.2)
Employment status	
Unemployed	460 (39.7)
Full time	207 (17.8)
Part time	393 (33.9)
Self-employed	100 (8.6)
Subject of study	
Social Sciences	264 (22.8)
Engineering and Technology	203 (17.5)
Arts and Humanities	172 (14.8)
Accounts, Business and Management	147 (12.7)
Life Sciences and Medicine	138 (11.9)
Natural Sciences	76 (6.6)
Cross-disciplinary	119 (10.3)
Other	41 (10.3)

### Psychosocial characteristics of the total sample

**Fig 1:**
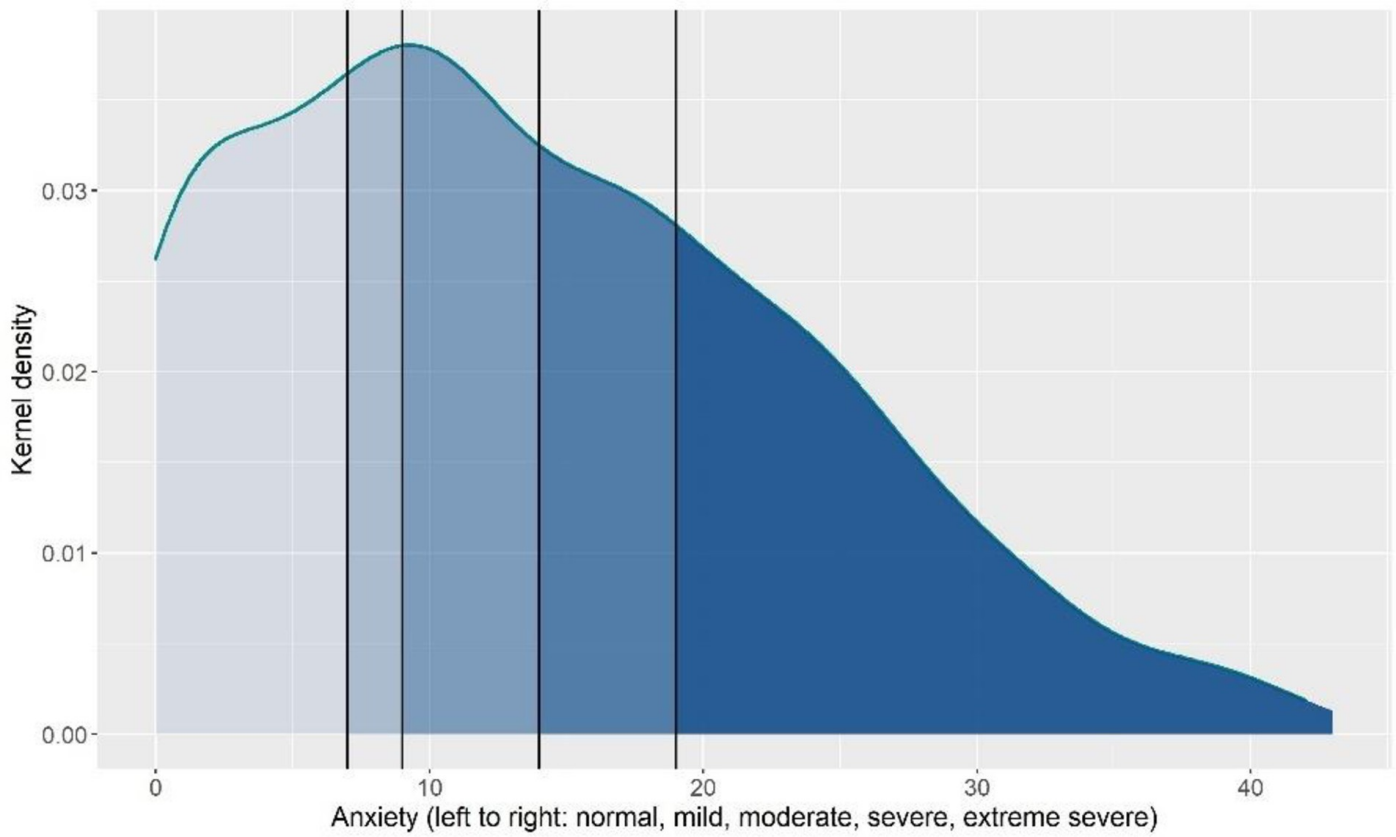
Distribution of anxiety.

**Fig 2:**
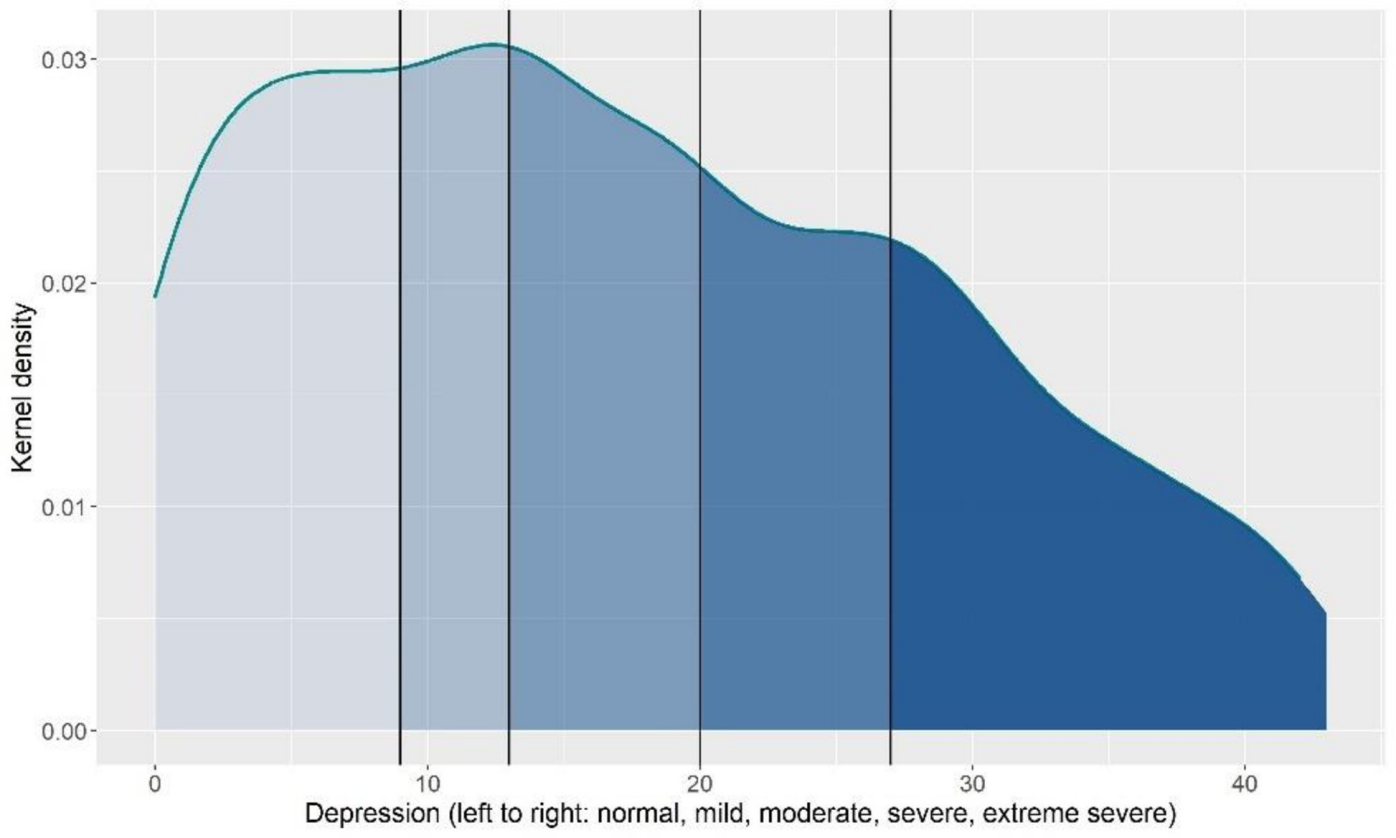
Distribution of depression.

**Fig 3:**
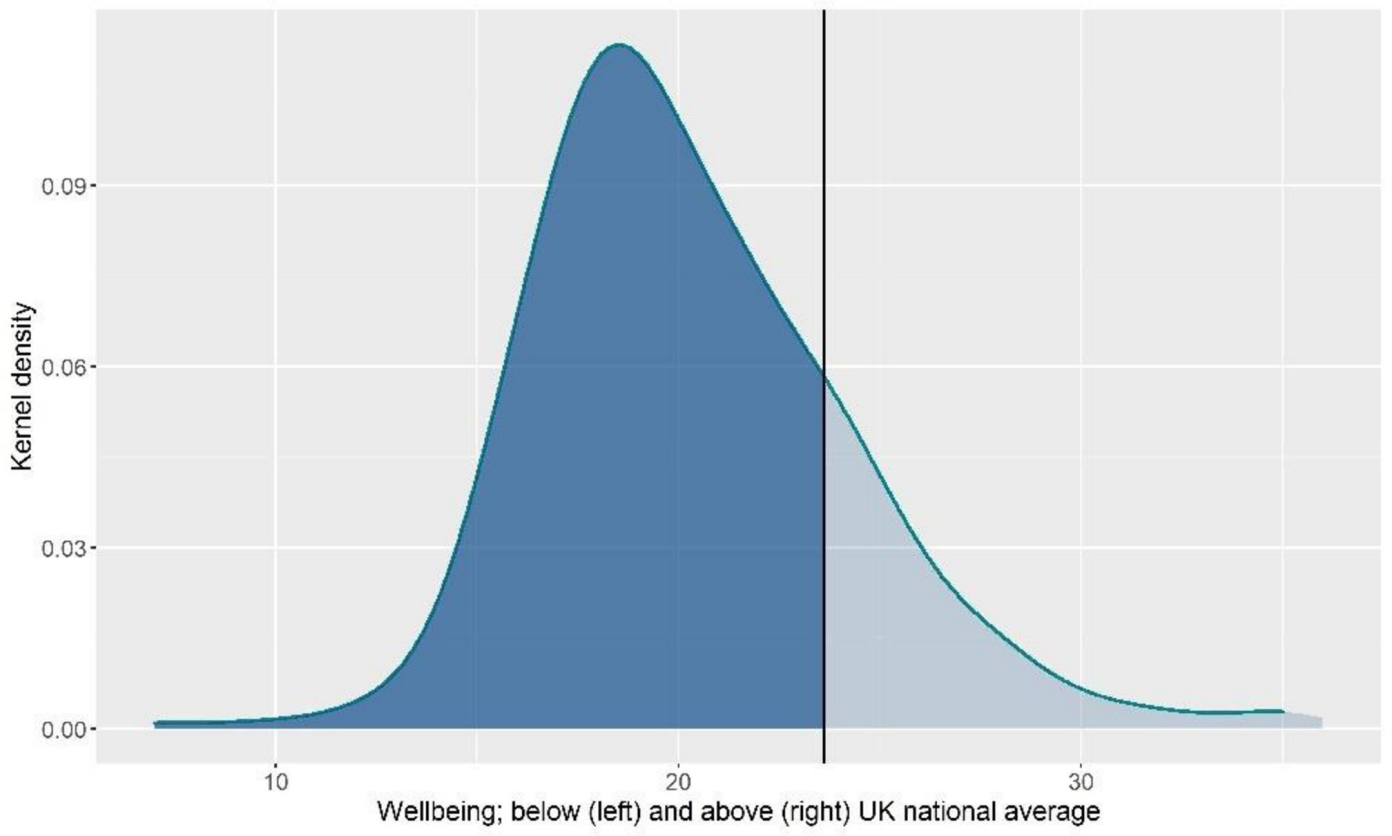
Distribution of wellbeing.

The average anxiety (M = 13.67, SD = 9.92, 63% moderate to extremely severe, Fig 1) and depression (M = 17.04, SD = 11.56, 58.2% moderate to extremely severe, Fig 2) scores were considered moderate. The average wellbeing score was 20.31 (SD = 3.93, Fig 3). Average general self-efficacy score was 28.29 (SD = 5.98) and coping strategies varied across the sample with students reporting a mean between 15 and 28 on the three coping styles; emotion focused coping had the highest mean (M = 27.59, SD = 6.37) and avoidant coping had the lowest (M = 15.77, SD = 4.69).

### Factors associated with mental health and wellbeing

The fitted models statistically explained 43.4% of the variance in anxiety, R^2^ = .434, F (43, 1116) = 45.72, p < .001, R^2^_*Adjusted*_ = .424; 50.2% of the variance in wellbeing, R^2^ = .502, F (43, 1109) = 51.69, p < .001, R^2^_*Adjusted*_ = .492 and 57.4% of the variance in depression, R^2^ = .574, F (43, 1116) = 76.21, p < .001, R^2^_*Adjusted*_ = .566, among students in the current sample.

**Fig 4:**
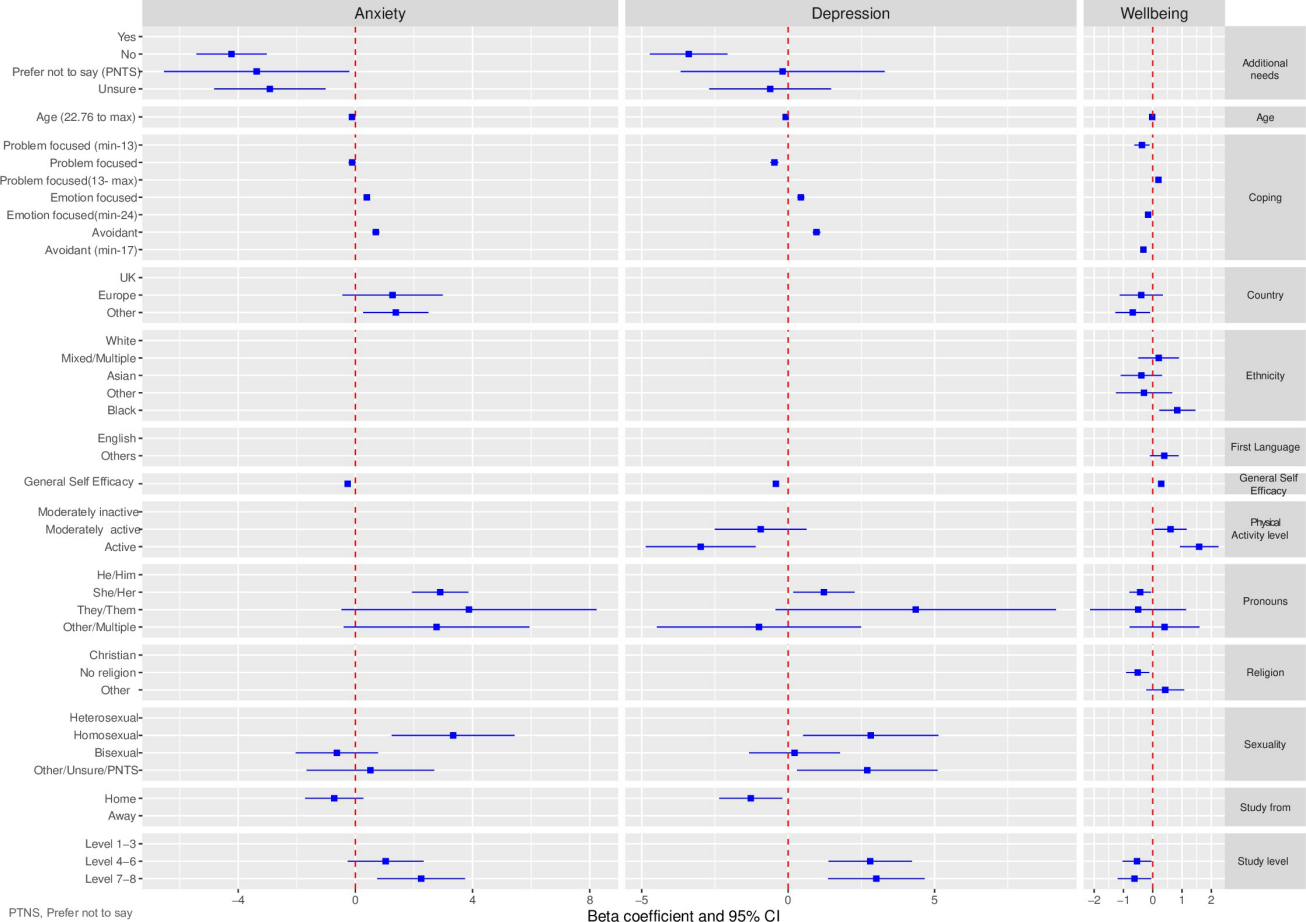


Fig 4 provides a graphical display of the associations between the predictor variables and the outcomes variables. The supplementary Information S1 File provides further statistical details of these associations. Compared to students self-identifying as he/him pronouns, students self-identifying as she/her pronouns were more likely to experience higher levels of anxiety and lower levels of wellbeing, while students self-identifying as they/them were more likely to experience higher levels of depression. Age was associated with anxiety and wellbeing but not depression. Younger students experienced higher levels of anxiety but better wellbeing. Ethnicity was significantly associated with wellbeing but not anxiety and depression. Students self-identifying as Black were more likely than White students to experience better wellbeing. Sexuality was only associated with anxiety. Compared to heterosexual students, students who self-identified as homosexual were more likely to experience higher levels of anxiety. Students with additional needs were more likely than students without additional needs to experience higher levels of anxiety and depression, but there was no significant association between having additional needs and wellbeing.

Compared to students studying at the pre-university level, students studying at the postgraduate level were more likely to experience higher levels of anxiety and depression. Students studying at the undergraduate level were also more likely than pre-university students to experience higher levels of depression. Education level was not associated with wellbeing. Location of study was significantly associated with anxiety, depression and wellbeing. Compared to students studying in the UK, students studying at non- European institutions were more likely to experience higher levels of depression and lower wellbeing. Students studying in any establishment outside of the UK were also more likely to experience higher levels of anxiety. However, students who considered themselves to be studying away from home were more likely to experience higher levels of depression.

Increased self-efficacy was associated with lower levels of anxiety and depression and higher wellbeing scores. Using more emotion-focused and avoidant coping strategies were associated with higher levels of anxiety and depression. Conversely using fewer emotion-focused and avoidant coping strategies were associated with higher wellbeing scores. Problem coping strategies were associated with lower depression and higher wellbeing scores but not significantly associated with anxiety. Students considered to be physically active were more likely than those who were moderately inactive to experience lower levels of depression and higher wellbeing.

## Discussion

This study examined the mental health and wellbeing of further and higher education students and the associating factors after returning to face-to-face (in-person) learning after Covid-19 restrictions.

### Mental health and wellbeing of students

The prevalence of anxiety and depression among students in this study was higher than some of the studies conducted during the pandemic [3, 5, 10], as well as pre-pandemic studies [57, 58]. A similar pattern was observed for wellbeing, with the current findings suggesting continued reduced wellbeing among students similar to during the pandemic [59] and an even lower average than the UK national norm in a pre-pandemic sample (20.31 vs 23.2, [42]). When compared to previous research, this finding is contrary to longitudinal studies which suggest subsiding mental health difficulties in the general population during or after Covid-19 restrictions, but consistent with the evidence that distress may persist among some students [26–30]. This confirms that although students may be allowed to return to in-person learning, it is possible that they are still experiencing mental health and wellbeing challenges associated with the pandemic. Another possible explanation could be the factors associated with academic success and the social environment at education settings [60]; however, there may be new or added factors that could be unique to specific groups of students.

### Students at risk of poorer mental health and wellbeing

Our findings align with some of the previous research suggesting that age, gender expression and pre-existing conditions can place some students at risk of experiencing poorer mental health and wellbeing [7, 11, 12]. Our findings also build on existing literature [13] suggesting that students who identify as gender non-conforming and/or students who identify as homosexual may continue to experience higher levels of mental distress than others. In terms of ethnicity, our findings did not coincide with research that suggested belonging to Black and Asian or other non-White populations were a significant predictor of poorer mental health [14]. A possible explanation could be that most of the students who identified as non-White were studying in countries where they were not considered as a minority ethnic group (e.g., South Africa, Mexico). However, students studying in non-European countries did experience poorer mental health and wellbeing which may better be explained by research from low-and middle-income regions or countries with more deprived areas [5, 61]. Therefore, it is possible different countries experienced different levels of severity of Covid-19 and corresponding restrictions, and consequently implemented different guidelines that allowed students to return to in person learning sooner than other countries.

Conversely, Black students were more likely than White students to experience better wellbeing. This is an interesting finding that raises further questions about the experiences of students who study in countries where they are considered as belonging to a minority ethnic group. In this same light, our findings align with previous research suggesting that students with additional needs generally experience poorer mental health. Recent studies suggest that the vulnerable population was greatly impacted by lockdowns and isolations and therefore it is possible that the impact could persist [31], suggesting that even more support or interventions are needed to support these students. Taken together the current findings contribute to the well-established literature that some marginalised groups (based on gender, sexuality and having a disability) are at greater risk of poorer mental health and wellbeing.

Although there are mixed findings suggesting that the level of study and type of programme was associated with levels of mental health and wellbeing [5, 62], this study adds that both undergraduate and postgraduate are more likely than pre-university students to experience higher levels of depression, and postgraduate students are more likely than pre-university students to experience greater levels of anxiety. This is consistent with previous research suggesting increased anxiety and depression among older students, sometimes due to additional caring and financial responsibilities [63]. Notably this was not replicated for wellbeing as level of study was not significant in the resulting model. Relatedly, students studying away from home also experienced higher depression levels compared to those who were studying at home, but this was not the case for anxiety and wellbeing. Although several qualitative and quantitative studies suggest that these mental health problems co-existed in young people during the early stages of the pandemic [3–10], it appears this may not be the case as students return to-in person learning. It is possible that factors associated with online learning and limited face-to-face support contributed to the onset of higher levels of anxiety. As for students studying away from home, studies suggest a greater risk of poor mental health among international students [64], but less is known about students who study in their home country but moved cities or provinces. This study accounted for various categories of students who study away from home which could explain any contradictory findings and thereby broaden our knowledge.

### Helpful coping strategies

Emotion-focused and avoidant coping strategies were associated with higher anxiety and depression and lower wellbeing while problem-focused coping was associated with lower depression and better wellbeing. Researchers with this belief suggest that problem-focused coping is more effective in controllable circumstances, but emotion-focused coping may be more effective in uncontrollable circumstances [65, 66]. Although these findings may be promising, there are mixed views on coping styles as there is also some evidence suggesting that in specific health conditions like SARS, all types of coping may act as a buffer against its negative impact [67]. Owing to the subjective nature of the experiences of returning to in-person learning, more qualitative evidence to explore if emerging themes like “focusing on positives” and “supporting each other” which were identified among female undergraduate students in the early stages of the pandemic, still persist [68].

In line with the extant literature, increased self-efficacy was associated with improved mental health and wellbeing [16]. However, some evidence from the first year of the pandemic suggested that although allied health students’ self-efficacy increased over time, their mental health did not improve [15]. Based on that finding, it is possible that allied health students may have experienced even more challenges than other programmes. Yet, these significant differences between programmes were not consistent in our study. Despite these contradictory findings, increasing self-efficacy among students have been found to be useful in improving mental health and wellbeing among students in higher education settings [69, 70]. This is an important finding that could contribute to the further exploration of self-efficacy and positive psychology as a key construct for promoting resilience in students [71, 72].

Lastly, our findings contribute to the growing body of evidence that physical activity positively correlates with better mental health and wellbeing among students. Our findings also add to this knowledge by suggesting that physical activity is associated to specific psychosocial problems like depression and wellbeing but not anxiety. The current finding also identified that more than 3 hours of physical activity per week of different activities (e.g., walking, cycling, gym) may be necessary in order to reduce symptoms of depression and improve wellbeing.

### Strengths and limitations of this study

Despite the potential contributions to knowledge, our findings and conclusions were interpreted within the context of some limitations. First, the study was based on self-reported questionnaires, which could have introduced some respondent bias. Second, the differences in Covid-19 restrictions across countries and at different times must be noted. Third, we used a modified measure of physical activity which has not yet been validated. However, the current version was endorsed as fit-for-purpose by a group of students and demonstrated adequate reliability within our sample. Therefore, we also acknowledge the potential benefits of student consultation and active participation throughout this study. Although the representativeness of the sample from different countries can be noted as a strength, students from some countries were missed or under-represented which could influence the generalisability of our findings. Similarly, as this study was conducted online, students without access to the internet and a digital device may not have participated. It is possible that these students may represents a low-income group of students who may also be at risk of poorer mental health and wellbeing outcomes. Another possible limitation is that students in our sample could have completed the survey at different time-points from the time they had returned to in-person learning which could have impacted recall. Notwithstanding these limitations, this study includes a large and internationally diverse group of students (N = 1160), and controlled for several individual, contextual and psychosocial variables.

### Implications for policy, practice and research

The current findings suggest a need for continued, and possibly increased, availability of mental health support and student-centred interventions to meet the needs of students who develop or continue to experience pre-existing mental health symptoms and poor wellbeing during, and following, the COVID-19 pandemic. The findings also highlight the high prevalence of and disparities in mental health and wellbeing among specific groups of students. Thus, education institutions and policymakers should continue to prioritize the provision of mental health services and implement student-centred and tailored interventions that focus on treating, preventing and promoting both mental health and wellbeing. The differences in factors associated with anxiety, depression and wellbeing suggest a need for continued research to fully understand how Covid-19 may have impacted different mental health and wellbeing symptoms–including among higher education students. Similarly, when compared to previous research the slower rejuvenation in wellbeing compared to anxiety and depression suggests that continued research is needed to monitor changes over time. This study may also provide a basis for qualitative research to identify support needs and preferences of specific groups of students and the further exploration of coping strategies practiced by students. To inform intervention development, the findings of this study suggest that the inclusion of techniques related to self-efficacy, physical activity and problem-focused coping can potentially influence positive outcomes.

## Conclusion

Students with additional needs and students studying at the postgraduate level may be at greater risk of reduced mental health compared to students without additional needs and those studying at the pre-university level. Some additional support may be needed for older students and students identifying as she/her or they/them, and homosexual. Similarly, some additional focus should be given to students studying away from home, and at non-European universities. The current findings can inform ongoing Covid-19 research, university policies and interventions to better support students as they continue to return to in-person learning after Covid-19 restrictions. Specifically, targeted interventions focusing on useful coping strategies and increasing self-efficacy and physical activity may be beneficial for students.

## Supporting information

S1 File (XLSX)

## Abbreviations

Covid-19: Coronavirus
